# Sleep-HD trial: short and long-term effectiveness of existing insomnia therapies for patients undergoing hemodialysis

**DOI:** 10.1186/s12882-020-02107-x

**Published:** 2020-10-20

**Authors:** Mark Unruh, Daniel Cukor, Tessa Rue, Kashif Abad, Maria-Eleni Roumelioti, Susan M. McCurry, Patrick Heagerty, Rajnish Mehrotra

**Affiliations:** 1 Division of Nephrology, Department of Internal Medicine, University of New Mexico, Albuquerque, NM USA; 2 Nephrology Section, New Mexico Veterans Hospital, Albuquerque, NM USA; 3 The Rogosin Institute, New York, NY USA; 4 Center for Biomedical Statistics, University of Washington, Seattle, WA USA; 5 Child, Family, and Population Health Nursing, University of Washington, Seattle, WA USA; 6 Division of Nephrology, Kidney Research Institute, University of Washington, Seattle, WA USA

**Keywords:** Insomnia, Hemodialysis, Cognitive behavioral therapy, Trazodone, Actigraphy, Telehealth, Computer-based telephone interview

## Abstract

**Background:**

Patients with end-stage kidney disease (ESKD) treated with hemodialysis (HD) experience many distressing symptoms. One frequently reported symptom is insomnia. There are unique issues about HD treatments and schedules that disrupt regular sleep/wake routines and possibly contribute to the high severity of insomnia. Despite evidence for broad-ranging health effects of insomnia, very few clinical trials have tested the efficacy of treatments for HD patients. Cognitive-behavioral therapy for insomnia (CBT-I) is a recommended first-line therapy but largely inaccessible to HD patients in the United States, partly because they commit considerable amounts of time to thrice-weekly dialysis treatments. Another important reason could be the logistical and reimbursement challenges associated with providing behavioral health care at the dialysis center. CBT-I delivered by telehealth can overcome barriers to access, but its efficacy has never been rigorously tested for these patients. Pharmacotherapy is the most widely used treatment for insomnia; however, some drugs presently used are unsafe as they are associated with a higher risk for death for HD patients (benzodiazepines and zolpidem-like drugs). The efficacy and safety of other medications (trazodone) for the treatment of insomnia has never been tested for patients treated with HD.

**Methods:**

This trial tests the short- and long-term comparative effectiveness of 6-week treatment with telehealth CBT-I, trazodone, or medication placebo. This will be accomplished with a randomized controlled trial (RCT) in which 126 participants treated with HD in community-based dialysis facilities with chronic insomnia will be assigned 1:1:1 to telehealth CBT-I, trazodone, or medication placebo, respectively; short-term effectiveness of each treatment arm will be determined at the end of 6-weeks of treatment and long-term effectiveness at 25-weeks. The primary and secondary patient-reported outcomes will be assessed with computer-based telephone interviewing by research scientists blinded to treatment assignment; additional secondary outcomes will be assessed by participant interview and actigraphy.

**Discussion:**

This clinical RCT will provide the first evidence for the comparative effectiveness of two distinct approaches for treating chronic insomnia and other patient-reported outcomes for patients receiving maintenance HD.

**Trial registration:**

NCT03534284 May 23, 2018.

**SLEEP-HD Protocol Version:** 1.3.4 (7/22/2020).

## Background

In the United States, most adults with end-stage kidney disease (ESKD) are undergoing long-term in-center hemodialysis (HD) in community-based facilities [1]. Approximately 40–85% of patients treated with HD have significant sleep disturbances [2–4]. A nationally representative study selected 1643 patients from 335 dialysis facilities in the United States using equal probability systematic random sampling. In this study, 50% of the patients had trouble falling asleep, 59% reported trouble with waking up during the night, and 49% reported trouble with early morning awakening, 53% reported one or more of these symptoms all or most of the time. A workgroup of the Kidney Health Initiative recently reported that patients undergoing HD treatment prioritized three physical symptoms for finding effective treatments for: insomnia, muscle cramps, and fatigue [5, 6]. In another study using a nominal group technique to obtain consensus, HD patients and their caregivers ranked sleep, fatigue, and anxiety/stress among the top 10 most important outcomes for clinical trials [7].

At least two factors may contribute to the high prevalence of insomnia in this patient population. First, day/night sleep reversal is a cardinal manifestation of uremia, the symptom complex associated with kidney failure. Studies have also shown that the severity of sleep problems are related to biochemical measures of kidney failure, suggesting insomnia may be the result of the accumulation of various uremic toxins [8]. Second, while HD is partially effective in removing uremic toxins, it also associated with poor sleep quality. Several studies have shown that compared with advanced kidney disease patients not treated with dialysis, patients treated with HD have a significantly shorter total sleep time and sleep efficiency and more significant sleep fragmentation [9]. This may be a direct result of unpredictable HD treatment schedules, such as early morning or late evening dialysis shifts, which make maintaining regular sleep/wake routines challenging. There is also evidence showing that half of all HD patients nap during the thrice-weekly dialysis treatments, inconsistently disrupting homeostatic sleep drive and circadian rhythm routines [10].

Insomnia is an essential contributor to other poor health outcomes in HD patients. Insomnia in dialysis patients is strongly associated with daytime sleepiness and worse quality of life [10]. Over 50% of dialysis patients report daytime sleepiness, fatigue, pain, and impaired quality of life, and over 25% have depression [3]. Successful treatment of insomnia in other patient populations has been shown to produce improvements in quality of life [11], but this has never been examined for patients treated with HD. Recent studies have also shown a higher adjusted risk for death in HD patients with poor sleep [7]. Importantly, the higher frequency of sleep disturbances in HD patients is independent of sleep apnea (an occlusion of the airway [obstructive sleep apnea], absence of respiratory effort [central sleep apnea], or a combination of these factors [mixed apnea]) and Restless Legs Syndrome (RLS), defined as an overwhelming urge to move legs, which is worst at rest and night and relieved by movement [4, 12]. In sum, it is evident that insomnia is a contributor to many highly prevalent symptoms and poor health outcomes in HD patients.

Notwithstanding the wide-ranging adverse effects of insomnia on the health and well-being of HD patients, no clinical trial has ever been done to determine the efficacy of any treatment for insomnia in HD patients in the United States. Short and Long-term Effectiveness of Existing insomnia therapies for Patients undergoing HD (SLEEP-HD) is the first multi-center randomized controlled trial (RCT) for treatment of insomnia in patients undergoing HD and seeks to test the comparative effectiveness of two treatments for insomnia in patients undergoing HD.

## Methods/design

### Study design and overview

**Fig. 1:**
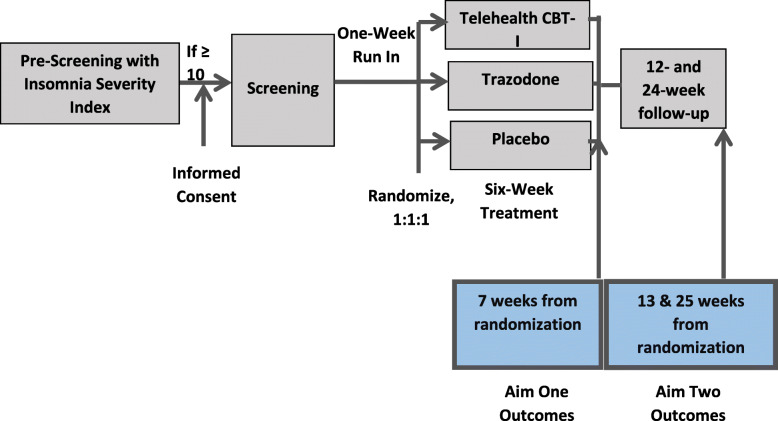
Study overview

SLEEP-HD is a parallel-group RCT wherein 126 HD patients treated in community-based dialysis facilities in Seattle and Albuquerque will be randomized 1:1:1 over 31 months to 6-week treatment with telehealth Cognitive Behavioral Therapy for Insomnia (CBT-I), trazodone, or medication placebo (Fig. 1). The investigators were considering increasing the sample size to 141 HD patients, but are in the process of re-evaluating this modification in co-operation with the NIH due to the adverse impact of the COVID-19 pandemic on all clinical research operations. SLEEP-HD has two specific aims designed to test two hypotheses:

- *Specific Aim One* – To compare the efficacy of 6-week treatment with telehealth CBT-I vs. trazodone vs. placebo for the treatment of chronic insomnia in patients undergoing HD. *Hypothesis One* - We hypothesize that the efficacy of six-week treatment with both telehealth CBT-I and trazodone will be superior to medication placebo in improving self-reported sleep.
- *Specific Aim Two* – To compare the sustained efficacy of the 6-week treatment with telehealth CBT-I vs. trazodone vs. placebo for the treatment of chronic insomnia in patients undergoing HD by examining outcomes at 25 weeks from randomization.
- *Hypothesis Two* - We hypothesize that telehealth CBT-I will be superior to both trazodone and placebo in improving self-reported sleep at 25 weeks from randomization.

The primary and secondary outcomes (Table 2) for the two aims will be ascertained at weeks 7 and 25 from randomization, respectively.

### Rationale for study interventions

#### CBT for insomnia (CBT-I)

CBT-I is the first line therapy for insomnia. In adults with insomnia, CBT-I improves sleep latency, wake after sleep onset, sleep efficiency, sleep quality, and can induce insomnia remission [21–40]. Based on this evidence, the American College of Physicians strongly recommends CBT-I as the first-line treatment for insomnia [41, 42]. These studies have limited relevance for HD patients as they have unique challenges to maintaining regular sleep/wake routines. It is unknown if CBT-I will be effective in favorably modifying these behaviors to improve sleep for this patient population. Telehealth, a two-way video interaction between the patient and the therapist, overcomes accessibility challenges and has been identified by the American Academy of Sleep Medicine as having the potential to narrow the gap between the availability of sleep providers and the number of patients needing insomnia treatment [43]. To date, only one center has reported a case series of its experience with telehealth CBT-I showing improvement; however, there was no control group to determine efficacy [44].

#### Trazodone

Pharmacotherapy is widely used as a first-line therapy for insomnia including HD patients; it is also an alternative for patients unwilling to engage with CBT-I or for whom CBT-I is unavailable or inaccessible. However, the drugs used widely have either concerns about safety or efficacy, or have been insufficiently studied for patients undergoing HD. Trazodone is an attractive option as population surveys show it to be the most common or second most common drug prescribed for insomnia in the United States for HD patients, yet is remarkably understudied [45, 46].

### Setting and participants

A total of 126 ESKD patients undergoing HD with chronic insomnia will be recruited from two clinical sites at the University of Washington, Seattle, WA and the University of New Mexico, Albuquerque, NM, which collectively have a wide range of dialysis clinics that vary in geographic area, size, and population served. These clinics were selected to ensure a diverse HD population with adequate representation of females and minority groups, including Blacks, Native Americans, and Hispanic/Latinos.

### Eligibility criteria

A) **Inclusion Criteria**

- Age 18 Years and older
- Undergoing thrice-weekly maintenance hemodialysis for ≥3 months
- Able to speak English
- Insomnia Severity Index (ISI) score ≥ 10 at pre-screening with sleep disturbances for ≥3 nights per week for ≥3 months [13].

B) **Exclusion Criteria**

- Severe depression assessed by Patient Health Questionnaire (PHQ)-2 and if appropriate, PHQ-9 [47]
- Suicidal Ideation
- Alcohol abuse on alcohol assessment questionnaire (score ≥ 2) or substance abuse on Drug Abuse Screening Test (DAST)-10 questionnaire (score > 5) [48, 49]
- Severe restless legs syndrome
- Treatment with trazodone in the past 1 month
- Known allergy to trazodone (self-reported or by chart review)
- Current treatment with monoamine oxidase inhibitors or in the preceding 14 days
- Current treatment with linezolid (self-reported or by chart review)
- Current treatment with other drugs that are inhibitors of CYP3A4 (e.g., itraconazole, clarithromycin, voriconazole), or known to prolong QT interval including Class 1A antiarrhythmics (e.g., quinidine, procainamide) or Class 3 antiarrhythmics (e.g., amiodarone, sotalol), antipsychotic medications (ziprasidone, chlorpromazine, thioridazine), and quinolone antibiotics
- Pregnancy, or lactation, or women of childbearing potential not willing to use adequate birth control
- Life Expectancy < 3 months
- Expected to receive a kidney transplant or transition to home dialysis (peritoneal dialysis or home HD) within 6 months
- Any other condition including cognitive impairment that, in the opinion of the investigator, should preclude patient participation in the clinical trial.

### Screening

Patients meeting inclusion criteria with ISI score ≥ 10 will be invited to sign an informed consent form to complete screening activities. The ISI is a 7- item instrument that measures self-reported insomnia. Each item is rated on a 0–4 scale with a total score range of 0–28. The instrument has been validated using both the classical test and the item response theory. After signing the consent form the following assessments will be completed: a) Medication Review to ascertain current use of trazodone or in the preceding 1 month; b) Patient Health Questionnaire-2 (PHQ-2); if the score on PHQ-2 is > 3, the PHQ-9 will be administered; c) Alcohol abuse on CAGE alcohol assessment questionnaire and DAST-10 for substance abuse; d) Cambridge-Hopkins RLS questionnaire; e) Medical history for co-existing illnesses; f) Pregnancy, or lactation, or women of childbearing potential.

After a final review of inclusion/exclusion criteria, eligible participants will be provided with instructions to complete sleep diaries for the run-in period which will also include the following procedures:

- The participants’ nephrologist will be informed about their enrollment in the clinical trial and if randomized to drug therapy, with a list of medications that could prolong QTc on EKG in people treated with trazodone, as listed in the United States Food and Drug Administration package insert for the drug;
- Placement of a wrist actigraph on the participant and provision of instructions on wearing the device (see section 4.3 below). The actigraph will be placed on the arm opposite to the upper extremity with the functional arteriovenous access being used for HD. In participants with a lower extremity arteriovenous access or with a central venous catheter, the actigraph will be placed on the non-dominant arm.
- The 7-day run-in period will start as soon as possible after the completion of the screening procedures and placement of the actigraph.

### Baseline visit

This will occur 7 days after the start of the run-in period and to the extent possible, on the first dialysis day of the week (Monday or Tuesday).

The following procedures will be completed at this visit:

- The actigraphy devices will be collected from the participants;
- The online or paper sleep diaries will be reviewed and/or collected;
- The dialysis shift days and the dialysis shift start time for each study participant will be noted;
- The case-report form on cumulative weekly use of sedatives/hypnotics and opiates will be completed, assessing the use of sleep aids for the preceding 7 nights.
- The participants will be scheduled to receive a phone call for the baseline Computer-Assisted Telephone Interviewing (CATI).

### Computer-assisted telephone interviewing (CATI)

An interviewer blinded to study intervention allocation will call the participant to initiate the CATI to complete the ISI (primary outcome measure) and seven pre-specified secondary patient-reported outcome measures (Table 2). CATI will occur at baseline, and 4, 7, 13, and 25 weeks from randomization.

### Randomization

The participants will be randomly assigned, 1:1:1 to CBT-I or trazodone or medication placebo using block randomization through a secure study web portal after the baseline CATI calls are completed, and no more than 25 days after completion of pre-screening with ISI. Participants will be randomly assigned to telehealth CBT-I, trazodone, or medication placebo, stratified by site using blocks of varying size [3 or 6] to ensure that the three groups are balanced at periodic intervals. The participants should begin treatment within 10 days from the date of randomization (first CBT-I session or provided with the first supply of medication).

## Study interventions study arms

### Telehealth cognitive behavioral therapy for insomnia

In this clinical trial, we will use telehealth CBT-I as a comparator and this will serve the dual purpose of testing the efficacy of CBT-I itself for HD patients, and its delivery via telehealth. Participants randomized to this arm will receive a treatment session once weekly for 6 weeks (Table 1) [51]. Each session is anticipated to last about 30 min. The content of each of these sessions will be adapted from standard CBT-I protocols to include discussion of factors unique to persons treated with HD that can increase insomnia risk such as napping during HD treatments, and inconsistent or very early or late dialysis treatment schedules [50]. The CBT-I sessions will be delivered by a trained CBT therapist face-to-face with the participant via a fully interactive HIPPA-compliant video telehealth platform, Zoom.

**Table 1:** Components of Telehealth CBT-I Intervention [50]

Week	Key Component	Session-Specific
**1**		Sleep changes with ESKD; rationale for approach; stimulus control instructions
**2**	Review Diary and Behavioral Sleep Plan	Sleep Scheduling/Bed Restriction
**3**		Sleep stages and cycles
**4**		Constructive Worry; Mindfulness/ Relaxation Exercise
**5**		Changing beliefs, attitudes about sleep/Sleep Hygiene
**6**		Maintenance/relapse prevention plan

#### Training and certification prior to implementation in trial

Each therapist will undergo in-person training by certified CBT-experienced study psychologists (DC, SMM). Following the completion of training, each therapist is required to complete mock interventions prior to implementation of the intervention in the clinical trial. These mock sessions will be audio recorded and will be reviewed by a study psychologist using a structured fidelity adherence form. Once the therapists are deemed competent in the intervention, they will be ready to implement the intervention in the clinical trial.

#### Monitoring the Fidelity of the intervention

During the conduct of the trial, weekly conference calls will be scheduled with the therapists during which clinical and implementation issues will be discussed, and cases reviewed. All individual CBT sessions will be audio recorded using the zoom platform. All CBT-I sessions for the first four participants assigned to each therapist will be reviewed to ensure the therapists are consistently following the CBT-I intervention protocol, and thereafter 2 sessions will be reviewed to ensure ongoing fidelity to the intervention. Review of audio recordings will be as timely as possible to provide meaningful feedback to therapists and feedback will be provided at the ongoing supervision calls or through email. This approach will allow therapists to receive support and real-time feedback to ensure the highest standards for delivering the behavioral intervention.

#### Monitoring adherence to intervention

Adherence to CBT-I will be monitored by recording number of completed sessions, duration of each session, and completion of other behavioral homework assignments (e.g., mindfulness exercises).

### Drug therapy

The site investigators will prescribe trazodone (50–100 mg) or corresponding medication placebo to the study participants randomized to the respective arms. The dose range selected for the trial (50–100 mg) is guided by prior studies with trazodone [52]. The starting dose for trazodone will be 50 mg, and if after the end of the first or second week of treatment the participants report inadequate improvement in sleep, they will have the option to increase the dose to 100 mg. The dose achieved by the end of week 3 will be maintained for the remaining 3 weeks.

#### Drug procurement and dispensation

Prior to launching the clinical trial, the Investigational Drug Services at Harborview Medical Center in Seattle, where the PI is based, will procure 50-mg tablets of trazodone from the same manufacturing stock for both clinical sites and prepare a matching placebo. The medications will be dispensed as per the protocol by the Investigational Drug Services at each site, and the study coordinators will hand the study drug to the participants at the dialysis facility when they arrive for their routine HD treatments; this will occur once during week 1 where a 3-week supply of the drug will be provided (with capacity to increase the dose to 100 mg starting week 2), and at week 4 where a 3-week supply of the drug for the dose being taken during weeks 4, 5 and 6.

#### Monitoring drug adherence

The participants will be asked to return the medication bottles for study drug at week 4 and week 7, and the coordinators will perform a pill count to assess adherence with the medication. All unused drugs will be destroyed by the Investigational Drug Supply.

## Ascertainment of outcomes

The summary score from the ISI at weeks 7 and 25 from randomization will be the primary outcome measures for Aims One and Two, respectively.

### Patient-reported outcomes

The primary ISI score and secondary patient reported outcomes (Table 2) will be ascertained by CATI calls at baseline, 4, 7, 13, and 25 from randomization by interviewers blinded to the study assignment. The baseline assessment will be performed upon completion of the run-in period and prior to randomization. CATI operators will conduct the assessments using scripts prepared for the call.

**Table 2:** Patient Reported Outcome Instruments Collected by CATI

**1.**	Insomnia Severity Index (ISI) [13]
**2.**	Pittsburgh Sleep Quality Index (PSQI) [14]
**3.**	Epworth Sleepiness Scale (ESS) [15]
**4.**	Functional Assessment of Chronic Illness Therapy Fatigue (FACIT-F) Scale [16]
**5.**	Graded Chronic Pain Scale (GCPS) [17]
**6.**	Patient Health Questionnaire-9 (PHQ-9) [18]
**7.**	Generalized Anxiety Disorder-7 scale (GAD-7) [19]
**8.**	Short Form 12-item health survey (SF-12) [20]

### Cumulative use of sedatives and hypnotics in prior week

This secondary patient reported outcome measure will be ascertained during the same week as the CATI call is completed, at baseline, and weeks 4, 7, 13, and 25 from randomization. The participants will be asked to report the number of nights during the previous 7 nights that they used a prescription, or over the counter or other sleeping aid(s) (including marijuana, if taken for sleep). This information will be supplemented by a review of the electronic medical records. The cumulative use of prescription opioids in the previous 7 days will be recorded similarly.

### Actigraphy

Actigraphy will be performed at three time-points: (a) the 1-week run-in period; (b) week 7 from randomization; and (c) week 25 from randomization. The key secondary outcome measure will be the average nighttime sleep efficiency (percent time asleep of total time spent in bed).The actigraphy data will be used to compute the following variables for additional exploratory analyses: a) Sleep onset latency; b) Total sleep time; c) Total wake time; d) Daytime inactivity; and e) Light exposure. Each assessment will last 7 days, will be scheduled to start and end with a regularly scheduled dialysis treatment, and the study coordinator will meet with the participant in his/her HD facility. Actigraphy data collection will be scheduled such that the 7-day period starts the week prior to the CATI and ends the week CATI is performed. The actigraphy data will be downloaded and scored using the Actiware 6.0.9 software (Phillips Respironics, Bend, OR) to determine wake and sleep based on one-minute epochs. The actigraphy scorers will be blinded to the randomized treatment assignment of the participant. The validity of data from actigraphy will be increased by our approach of asking participants to maintain sleep diaries to guide scoring of bed and rising times, and to examine artifacts in data collection, e.g., removal of the device [53]. Additionally, light sensors and actigraphy event marker data will be used in conjunction with the diaries to ensure that in-bed and out-of-bed (daytime) periods used for calculating sleep parameters are accurate.

## Treatment

### Telehealth CBT-I sessions

These visits (Table 3) will occur once a week for 6 weeks via telehealth using the Zoom platform delivered by a therapist. For any given participant, the same therapist will administer each of the 6 CBT-I treatments. The sessions will be held with the participant either (a) at the dialysis facility (while undergoing HD or at another time), using a personal device (tablet or lap-top) or one provided by the research team; (b) or at home using a personal device. Prior to each session, data from the sleep diaries will be over the telephone by either the study coordinator or the therapist. All CBT-I sessions will be audio-recorded with the permission of the participant.

**Table 3:** Study Procedures Timeframe

	Pre-Screen	Screen	Run-In		Treatment						Follow-up from Randomization		
WEEK			-1	0	1	2	3	4	5	6	7	13	25
**Insomnia Severity Index**	x			x				x			x	x	x
**Structured Interview, Inclusion/Exclusion Criteria**		x											
**Randomization**				x									
**CBT-I Telehealth Sessions**					x	x	x	x	x	x			
**Trazodone/Placebo Visits**					x	x	x	x		x			
**Treatment Adherence Assessment**								x			x		
**CATI**				x				x			x	x	x
**Actigraphy with sleep diary**			x							x			x
**Concomitant Sedatives/Hypnotics**				x				x			x	x	x

### Medication visits

For participants randomized to medication (trazodone or placebo), the visits and related procedures will be identical. Medication visits will occur at weeks 1,2,3,4 and 6.

Visit one will occur, to the extent possible, on the day of the first dialysis treatment of the first week following randomization. The participants will be provided with a three-week supply of 50-mg trazodone or matching placebo (Table 3).

Visit two and three will occur on the day of the first dialysis treatment of the second and third week respectively following randomization and 1 week following the “Medication Assessment Visit One and Two”. The coordinator will ask the participant if they have been prescribed any new medications since the last visit to screen for contra-indicated drugs to prolong QTc interval on the EKG. The participants’ response to drug therapy will be assessed and they will be advised to either continue on the 50 mg dose if they are satisfied with their sleep over the previous 7 days, or to increase/decrease 50–100 mg based upon problems/tolerability with sleep in the previous 7 days.

Visit four and five will occur on the day of the first dialysis treatment of the fourth and sixth week respectively following randomization.

### Early closeout

For participants that choose to terminate participation in the study, all efforts will be made to collect information about cumulative use of sedatives and hypnotics and opioids in the previous 7 days, and an early closeout CATI call will be scheduled for collecting data on primary and secondary patient reported outcome measures.

## Adverse events

### Potential adverse events from study interventions

1. Adverse Effects from CBT-I: The risk classification for this arm is “not greater than minimal.” The greatest risk with CBT-I is discomfort/fatigue associated with restricting time in bed (including reducing daytime napping); these symptoms are usually temporary but will be monitored closely given their overlap with post-dialysis symptoms. Additional potential risks include breach of confidentiality which is inherent to any telehealth intervention regardless of the participant’s location.
2. Adverse Effects from Trazodone:

- With drug-drug interaction trazodone could prolong QTc interval on the EKG
- Side effects (> 5%) reported more frequently than with placebo are (i) drowsiness; (ii) nervousness; (iii) dizziness; (iv) fatigue; (v) dry mouth; (vi) nausea; and (vii) vomiting
- Priapism is another possible but extremely rare side effect

3. Psychological discomfort from completing patient-reported scales
4. Loss of patient confidentiality

### Anticipated adverse events in the hemodialysis population

Patients undergoing HD experience many adverse events from their underlying health, co-existing illnesses, and concomitant medications. These adverse events include: (1) death; (2) fluid overload; (3) congestive heart failure; (4) vascular access events, such as thrombosis or infection or dysfunction; (5) atherosclerotic cardiovascular events; (6) infections, such as pneumonia; and (6) laboratory abnormalities such as anemia, hyperphosphatemia, and secondary hyperparathyroidism.

### Monitoring for adverse events

The participants will be monitored for the occurrence of adverse events as:

- The informed consent document will provide the name of a study staff person with a phone number to be contacted in the case of an emergency or if an adverse event occurred outside the time frame of study visits;
- Study staff will evaluate the participants 5–6 times during the 6-week treatment period either via tele-health, telephone, or in-person; and.
- Participants will be withdrawn from treatment if they experience a serious adverse event directly attributable to the study intervention and, in the opinion of the site investigator, if the treatment cannot be safely reinstituted.

### Reporting of adverse events

All adverse events experienced by study participants from the time of registration into the study (randomization) will be recorded and summarized by random assignment group. The summary will be submitted to oversight groups at periodic intervals.

All serious or unanticipated adverse events will be reported by each of the clinical sites to the Data Coordinating Center within 24 h of becoming aware of these events using a structured reporting form. Each site will also be expected to follow local reporting policies such as to the Institutional Review Board. Serious adverse events are the ones that result in death, are life-threatening, lead to prolonged hospitalization, or result in disability or incapacity. Unanticipated adverse events are unexpected adverse events, which in the opinion of the investigator, could reasonably be considered associated with participation in the research study. The Data Coordinating Center will be responsible for communicating these to the Data Safety and Monitoring Board consistent with the policies agreed upon and outlined in the Board’s charter.

## Statistical analyses

### Statistical analysis for aim one

The primary outcome for Aim 1 is the ISI measured at 6 weeks after initiation of treatment. Given that we have three arms, a simple analysis for this aim would be an ANOVA analysis. However, to maximize the use of information and to account for potential loss to follow-up and/or intermittent missing outcome measures, we will use a comprehensive longitudinal analysis to make inference regarding group differences across time. Specifically, we will use linear mixed models for the analysis of all outcomes including the baseline, 4, 7, 13 and 25-week ISI measurements [54]. For longitudinal models we will use indicator variables to model the 4, 7, 13, and 25-week changes from baseline (control group temporal trend) and then use two sets of treatment-by-time interactions to characterize the difference in the change over time for the CBT-I group compared to placebo, and the trazodone group relative to placebo. Our primary analysis will use linear mixed model estimation assuming an unstructured outcome covariance matrix in order to minimize model assumptions. Given randomization, we know the baseline group mean difference is zero and therefore, an appropriate longitudinal model imposes this known baseline constraint. The primary test for Aim 1 is a longitudinal variation on ANOVA that would simultaneously test that both the CBT-I treatment by 7-week interaction, and the trazodone by 7-week interaction are zero. This null hypothesis assumes that the change from baseline to 7-weeks is the same for each of the three treatment groups. Secondary analysis will estimate the confidence interval for the pairwise comparisons of each of the three groups from the longitudinal model. A similar analytic approach will be taken for analyses of data for each of the nine pre-specified secondary outcomes. Exploratory analyses will be undertaken to determine if there is preliminary evidence for effect modification by gender, race/ethnicity, restless legs syndrome, cumulative use of sedatives/hypnotics or opiates, dialysis shift, and treatment adherence to CBT-I or trazodone.

### Statistical power for aim one

The standard deviation for ISI in large clinical trials in other populations has ranged from 3.8–5.6 [24, 35]. Based on these studies, we assume that the standard deviation of the 6-week ISI will be between 4.0 and 6.0 and we conservatively use 6.0 as the basis of sample size estimation. The minimal clinically important difference for this scale is between 6 and 8 for indication of an individually successful response to treatment [11, 13]. We ultimately targeted a group difference of 4.0 as the alternative that we would power our study to detect [11, 13]. Using simulation methods and assuming a correlation of 0.50 for baseline and follow-up measures we determined that 110 participants evaluated at 6 weeks is sufficient to obtain > 90% power to detect a 4.0 difference in ISI scores between the treatment groups combined and the placebo group. In order to have 110 evaluated participants we would need to inflate the enrolled sample size to account for an anticipated 10% loss to follow-up and arrive at our final sample size of *n* = 126.

### Statistical analysis for aim two

The primary outcome for Aim 2 is the ISI measured 25 weeks from randomization. The analysis will leverage the same longitudinal model fit for aim 1, with a parallel primary hypothesis test and confidence interval estimation at 25-weeks as at 7-weeks. Secondary analysis will estimate the full trajectory over time for each treatment group and allow comparison of the group longitudinal profiles. A similar analytic approach will be taken for analyses of data for each of the 9 pre-specified secondary outcomes. Exploratory analyses will be undertaken to determine if there is evidence for effect modification by gender, race/ethnicity, presence of restless legs syndrome, concomitant medications, and treatment adherence.

### Statistical power for aim two

We have based our sample size on Aim 1, yet we retain strong power to detect long-term treatment effects. A power analysis using a simple pre-post analysis for 25-week outcomes controlling for baseline is a conservative estimate of the power for the longitudinal model since the simple analysis would only use 25-week complete cases. If we assume 20% loss to follow-up through 24 weeks, then we would have 100 evaluated participants. For Aim 2 we would have 90% power to detect a CBT-I treatment effect of 4.0 points relative to trazodone and placebo and within the range of moderate treatment effects defined by standardized group differences of 3.0–4.8 points.

## Discussion

Insomnia is a common and distressing symptom for patients on HD, and there is evidence for a much larger impact on the health of patients. The SLEEP-HD study is an open-label RCT to compare two types of treatment for insomnia in participants who have ESKD on HD with chronic insomnia. The two types of treatment involved in the study are CBT-I or treatment with a drug (trazodone vs placebo).

CBT-I is the first line therapy for insomnia but is largely inaccessible to dialysis patients [22, 41]. Two clinical trials on HD, one from Taiwan with 72 patients and one from China with 103 patients, have demonstrated improvement in sleep quality, assessed with the Pittsburgh Sleep Quality Index, with group CBT-I [55, 56]. However, these trials cannot inform clinical practice in the United States where patients must commit 4 h thrice weekly to HD treatments in stand-alone dialysis facilities away from hospitals and medical facilities, making in-person CBT-I inaccessible. In addition, the most important reason CBT-I therapy is inaccessible to most people in the United States and not just HD patients is the lack of trained providers particularly in regions without specialty sleep centers. Thus, it is imperative to develop innovative solutions to make treatment for insomnia more widely available, including for those undergoing HD. Some treatment approaches have been tested in other groups without kidney disease such as self-help strategies, internet programs, or over the telephone [51, 57–60].These approaches introduce newer limitations as they don’t involve a face-to-face interaction with a therapist; internet-based CBT-I is modestly effective self-help therapy in the short run for insomnia [60], and telephone-based CBT-I has been shown to be efficacious as a first line treatment in healthy midlife women with insomnia symptoms (one of the four MsFLASH trial that was telephone delivered and focused explicitly on treating sleep disturbances) [61].

As for pharmacotherapy, there is limited evidence to support current practices for insomnia for dialysis patients. Notwithstanding clinical practice guidelines, pharmacotherapy is widely used as a first-line therapy for insomnia including HD patients; it is also an alternative for patients unwilling to engage with CBT-I or for whom CBT-I is unavailable or inaccessible. However, the drugs used widely have either concerns about safety or efficacy, or have been insufficiently studied for patients undergoing HD. Benzodiazepines and non-benzodiazepine benzodiazepine-receptor agonists such as zolpidem are prescribed for 8–26% of HD patients [62, 63] even though two cohort studies have shown that HD patients treated with these drug classes have a significantly higher risk for death [45, 63]. Trazodone is an attractive option as population surveys show it to be the most common or second most common drug prescribed for insomnia in the United States for HD patients, yet is remarkably understudied [45, 46, 64, 65]. The drug is metabolized in the liver into inactive metabolites and no dose adjustments are necessary with kidney disease. However, there are limited data to guide clinical practice. Low-quality evidence suggests its efficacy for treating primary insomnia; in two placebo-controlled trials, treatment with trazodone lasted only 7–14 days, and the only trial testing its efficacy as add-on to CBT-I enrolled just 20 patients [66–68]. Studies have shown the efficacy of trazodone for insomnia in patients with depression, Alzheimer’s disease, and alcohol/opiate dependence [68–75]. However, no studies have tested its efficacy and safety in the setting of partial correction of uremia as with HD and none are underway. Furthermore, HD patients have unique behavioral and scheduling challenges and have greater risk with daytime sleepiness when overlaid upon hemodynamic fluctuations with HD and frailty. This study will generate much-needed evidence to support or refute its widespread use for treating insomnia in HD patients.

There is a compelling need to identify effective treatments for insomnia in HD patients, and the interventions being studied in this clinical trial (telehealth CBT-I and trazodone) have a strong scientific premise. Most HD patients have significant impairments in quality of life, mainly from the high frequency of disabling symptoms. Insomnia is one of the most frequently reported symptoms, and studies of HD patients and other populations suggest that it is a significant contributor to other common symptoms and poor health outcomes. It is essential to know that insomnia is common in HD patients with comorbid depression but is independent of sleep apnea and restless legs syndrome [4, 12] . There are unique contributors to chronic insomnia in HD patients, and these include the biologic effects of residual uremia after partial correction, as is achieved with current dialysis technology, maladaptation to treatment schedules, and patients’ napping during treatments. If telehealth CBT-I is effective for treating insomnia in HD patients, it will create an option that is presently unavailable to patients. Trazodone is widely used but the data on efficacy for treating insomnia in HD patients are limited as well.

## Conclusion

SLEEP-HD is the first multi-center RCT for the treatment of insomnia in patients undergoing HD and seeks to compare the efficacy of telehealth CBT-I vs. trazodone vs. medication placebo for the treatment of chronic insomnia in patients undergoing HD.

Dialysis is a life-sustaining treatment for patients with kidney failure. Nevertheless, most patients experience many troubling symptoms, and disturbances in sleep are among the most reported problem. However, there has been limited research done to identify what treatments are effective for patients undergoing HD with sleep problems. Moreover, HD treatments are scheduled three times every week, making it extremely difficult for patients to have the time to receive cognitive behavioral therapy – a treatment effective in improving sleep in individuals without kidney failure. In this study, we plan to test the short- and long-term effectiveness of cognitive behavioral therapy with a therapist face-to-face delivered over the web, instead of in-person, with a drug. This study has the potential not only to benefit patients treated with HD but other patients that have difficulty in accessing treatment for sleep problems such as residents of rural and underserved areas.

## Data Availability

The data that support the findings of this study may be available on request from the PIs (R Mehrotra).

## Abbreviations

ESKD: End Stage Kidney Disease
HD: Hemodialysis
CBT-I: Cognitive Behavioral Therapy – Insomnia
RCT: Randomized Controlled Trial
ISI: Insomnia Severity Index
PHQ: Patient Health Questionnaire
DAST: Drug Abuse Screening Test
CATI: Computer Assisted Telephone Interviewing
PSQI: Pittsburgh Sleep Quality Index
ESS: Epworth Sleepiness Scale
FACIT-F: Functional Assessment of Chronic Illness Therapy – Fatigue
GCPS: Graded Chronic Pain Scale
GAD: Generalized Anxiety Disorder
SF − 12: Short Form – 12
